# Progress in Surgery

**Published:** 1900-04-21

**Authors:** 


### Progress in Surgery.

### SURGERY OF THE GALL-BLADDER, SPLEEN, AND PANCREAS.

**Cholelithiasis.**—Hunner[1] thinks there is a growing belief that gall-stones are in most cases of infectious origin. Naunyn first formulated this theory, and Cushing supported him. The latter proposed the following theory regarding the gall-stone formation in cases of typhoidal cholelithiasis: 1. The bacilli during the course of typhoidal infection quite constantly invade the gall-bladder. 2. The organisms retain their vitality in this habitat for a long period. 3. In the course of time the bacilli are almost invariably found clumped in the bile, suggesting the occurrence of an intravesical agglutinative reaction. 4. The clump presumably represents nuclei for the deposit of biliary salts, as micro-organisms may with regularity be demonstrated in the centre of recently-formed stones. 5. Gall-stones being present in association with the latent, long-lived infective agents, an inflammatory reaction (cholecystitis) in the viscus of varying intensity may be provoked at any subsequent period. In connection with these observations Hunner[2] reports a case of acute suppurative cholecystitis, with isolation of the bacillus typhosus, eighteen years after an attack of typhoid fever. He further refers to the experiments of Mignot, who states that the chief factors in the production of biliary lithiasis are: 1. The presence in the gall-bladder of an attenuated organism of any variety whatever. 2. A relative inertia of the biliary reservoir, causing partial stasis, and preventing the premature expulsion of soft concretions of cholesterin. Maclagan and Treves[3] report three cases, in which movable kidney produced all the symptoms of gall-stones, though no cholelithiasis was present. The symptoms included recurrent attacks of hepatic colic and jaundice. Newbolt[4] relates a successful case of cholecystotomy, in which the matting together of the omentum formed a tumour which quite obscured the gall-bladder, and prevented the gall-bladder from being brought to the surface.

**Splenectomy.**—Burtz[5] attributes the improved mortality after this operation not only to better technique and asepsis, but also to recent limitations in its field. After laceration the prognosis is relatively good. In malignant tumours and in wandering spleen operation is indicated, but the prognosis, especially when the tumour is very large, is not so good. Extensive adhesions and leukæmia of the spleen contra-indicate operation. In the former case the surface bleeding can hardly be arrested by tampon; in the latter the vessels of the pedicle are always large, but their walls are brittle and easily torn, the ligature cuts into the walls, and the patient succumbs to secondary hæmorrhage. Janz[6] also believes that operations on leucocythæmic spleens are unjustifiable, but says it is otherwise with pseudo-leukæmic spleen (idiopathic hypertrophy, anæmia splenica, primary splenomegaly or simple hypertrophy). Janz gives past results of splenectomy for this disease as 33 cases with 19 deaths according to Ceci, 19 cases with 11 deaths according to Orecchia, one successful case reported by Lennander, and one case of his own which lived over two months after splenectomy. Stone[7] records a successful case of splenectomy for floating spleen with twisted pedicle. Max Madlener[8] reports an operation for intestinal obstruction on a patient on whom he had performed splenectomy for rupture five months before. The patient made a good recovery. He tabulates 11 cases of excision of the spleen ruptured without any external wound, of which 8 recovered and three died. Max Madlener may claim, it appears, the first operation on a spleenless patient.

Trendelenburg[9] records six cases of rupture of the spleen, with two recoveries after operation. Probably the majority of cases of severe rupture of the spleen die in the first hour. The diagnosis is based upon: History of injury. After injury the patient may be able to walk or drive for half-an-hour, or even more. Then there is a feeling of acute pain in the splenic region, and a sense of extreme weakness. On examining the splenic region there is rigidity, tenderness, and dulness on percussion. There is pain on deep inspiration, the breathing is short and jerky, and a fracture of the ribs may be suspected. Pain spreads over the abdomen; abdominal rigidity and distension become apparent, especially in the upper left quadrant of the abdomen. Symptoms of hæmorrhage now develop rapidly, pallor, extreme anxiety, thirst; small, frequent pulse, and vesical tenesmus. The chance of success lies in an early operation.

**Tumours of the Pancreas.**—Zoja[10] concludes that as far as primary carcinoma of the head of the pancreas is concerned, there is no proper or constant symptomatology; the symptoms vary so much according to the position and extension of the tumour. The cases may be divided into three chief types: 1. Where the tumour takes a postero-inferior direction, causing compression of the vena cava, implicating the right suprarenal capsule and the retroperitoneal glands. 2. Where it takes an upward direction, causing symptoms of occluded bile duct or pyloric stenosis. 3. Where development takes place to the right, giving rise to occluded bile or pancreatic ducts—perhaps most common of all. Tumour may be felt; it depends on the situation, &c. Pain is not a constant symptom; melæna or hæmatemesis is a fairly common termination. The author never observed glycosuria in his cases, and attaches no importance to the search for indican.

**Traumatic Pancreatic Cyst.**—Van der Laan[11] records the case of a boy, aged 9, who, four months after a blow in the epigastrium developed a fluctuating tumour in the epigastrium, with the stomach resonance above it, and the tympanitic colon below. The tumour was tapped, but this operation was followed by pain, vomiting, and fever. On opening the abdomen it was found that the stomach had been wounded. This was sutured. Three weeks later, when the cyst had refilled, the old wound was opened, the cyst brought to the surface and drained. One tube was removed in five days, the second at the end of three weeks, after gradual shortening. At the end of six weeks the fistulous track had completely closed. The peril of tapping, even with precaution, is manifest in this case. Van der Laan notes that his case is the first instance of traumatic pancreatic cyst in a child.

[1] Amer. Jour. Med. Sci., Dec. 1899, p. 722; and Johns Hopkins Hosp. Bull., Aug.-Sept., 1899. [2] Loc. cit. [3] Lancet, Jan. 6, 1900, p. 15. [4] Brit. Med. Jour., Jan. 20, 1900, p. 142. [5] Ibidem, Nov. 25, 1899, Epitome, No. 409; and Deut. Med. Woch, Nov. 2, 1899. [6] Brit. Med. Jour., Oct. 28, 1899, Epitome No. 330; and Beitr. zur Klin. Chir., 1899. Vol. xxiii. pt. 2, [7] Annals of Surg, Sept., 1899, p. 321. [8] Brit. Med. Jour., Dec. 2, 1899, p. 1,567; and Munch. Med. Woch, Oct. 24, 1899. [9] Brit. Med. Jour., Dec. 9, 1899, Epitome No. 444; and Deut. Med. Woch, Oct. 5, 1899. [10] Brit. Med. Jour., Dec. 9, 1899, Epitome No. 439; and A. Policlinico, Aug. 1, 1899. [11] Brit. Med. Jour., Oct. 14, 1899, Epitome No. 297.

### GENERAL SURGERY.

(*Continued from page* 343, *Vol. XXVII.*)

**Fractures and Dislocations.**—One of the most admirable expositions of the modern and proper treatment of so called simple fractures is to be found in Mr. Marmaduke Shield's Lecture,[7] which should be read by all who take an interest in these cases. He emphasises the fact that a broken thigh or leg must be treated, perhaps, in a rough district, with poor appliances, with unskilled nursing, in an alcoholic and unruly patent. Truly such cases are amongst the most anxious trials of a medical life, a badly united or non-united fracture in such persons may serve as evidences of supposed want of skill for many long years—perhaps for the rest of life. The treatment of a fracture does not cease when the patient leaves the hospital, and unpleasant truths about these injuries can always be learned in the after progress if inquiries be made. The final results in private practice, on the other hand, is ever before the practitioner. It is nearly time the surgery of fractures was rewritten. The X-rays have marked almost a revolution in their diagnosis, and are a valued aid in their treatment. The first duty, therefore, is to obtain a skiagraph, and if the case occurs in a remote country district it is best to send the patient to the nearest public hospital, if he cannot afford the services of an expert, with the requisite apparatus. The ultimate results in a bad "Potts" fracture, in fracture of the upper or lower end of the femur, in fractures into the elbow joint, especially in old or rheumatic persons, are often very unsatisfactory. Some deformity, some lameness, some loss of mobility of the articulation persists, and may persist permanently, even with the best of skill and care. A man will break his thigh or leg in the worst possible manner, and he cannot be made to understand that the subsequent deformity or lameness is due to the severity of his injury, and not to lack of skill in treatment. It is Mr. Shield's practice to write an opinion, as in the following letter by way of example, appropriate to the case: "Dear Mr. ——,—The skiagraph of your injury shows that you have a fracture into the elbow joint of a complicated nature. I regret to have to say that when the reparative material is thrown out and new bone is formed, the future functions of the joint must be impeded, and stiffness and loss of motion will be the consequence. I will do all in my power to obviate this by proper and persevering early movements of the joint, but recovery will be difficult and tedious, and some impairment of usefulness must ensue; it is impossible now to say to what degree. I tell you this because, if you or your friends should desire some surgeon of repute to see your case, and advise concerning it, now is the time before the bones have joined. I should be sorry to receive blame afterwards for what is really unavoidable from the nature of your injury, and, if you are at all in doubt about what I say, you had better call in additional aid. Please keep this letter by you.—I am, dear sir, &c." Mr. Edmund Owen[8] also insists upon the difficulties of treating many fractures without some resulting deformity. Although a medical man may, in the management of a fracture, take every necessary precaution, and may treat the case with a "fair, reasonable, and competent degree of skill," the result may not satisfy his patient, and thus he is liable to the annoyance and expense of a civil action, and may be cast in damages. He advises, therefore, that every practitioner shall insure against such contingencies to the best of his ability in the way he suggests, viz., by getting a *confrère* to administer an anæsthetic, if necessary, in order that he may make his inspection absolutely complete. If he is in doubt about any injury; if he is not sure as to the line of treatment; if he wishes to relieve himself of some of the inevitable responsibility, he should certainly get some *confrère* to see the case with him. If still there is doubt, he should propose that the help of a radiographer be sought. The *Lancet*,[9] in an article on the Röntgen rays in surgical work, makes statements which have been subsequently disputed by well-known radiographers. It says that, so far as fractures are concerned, it must be admitted that the practical gain by their help is quite inconsiderable. No doubt in some cases our knowledge of the exact nature and extent of the fracture has been increased, especially in injuries to the wrist, knee, and ankle. In Colles's fracture, to take an example, it is now positively demonstrated that the line of fracture is rather lower than was generally thought, that the carpus preserves its relationship with the radius, that comminution of the lower fragment is common, that associated fracture of the styloid process of the ulna, though occurring, is not usual, and that the amount of the backward displacement of the carpus is by no means so great as we had, from clinical examination, been led to believe. Our knowledge is certainly more precise, but our capacity for effectively dealing with these fractures has not been increased to any appreciable extent. The writer of an article in the *New York Medical Journal*[10] is of opinion that in many instances the Röntgen Ray examinations are utterly uncalled for. He believes that, in view of the injuries that sometimes result, it is wrong to resort to the method unless there is a reasonable presumption that some important feature of the case will be revealed that would otherwise escape recognition, and his belief is that such cases are comparatively rare. Unfortunately, the point has been raised in at least one malpractice suit that the surgeon was guilty of neglect, because he had not made an X ray examination. But he urges the profession to repudiate this proposition, if the prospect is that it will be held to be generally applicable in cases of fracture. On the other hand, the well-known authority, Mr. Mansell Moullin,[11] states that the risk of causing injury has altogether disappeared. Many operators can point to hundreds of cases which they have taken, not one of which have ever suffered the slightest harm. He goes on to say that, as might be expected, the largest proportion and the most striking cases from the use of the Röntgen Rays have been furnished by the injuries and diseases of bones and joints. Those only who have experienced the difficulty of determining whether a fracture or a dislocation, or both together, may not be present in the neighbourhood of such a joint as the elbow when the soft tissues around are so swollen that no bony prominence can be felt can realise the immense help given by a well-lit fluorescent screen. Half a minute exposure is now enough. The nature of the injury is apparent at once, and, what is even more valuable, it is no less easy to ascertain whether the fracture is properly set or the dislocation completely reduced. The records of the past year have shown that the screen is of service to surgeons by enabling them to make sure at a glance that the bones are in their proper relative situation without touching the splints or giving the patient a moment's pain. Nelson[12] recommends suturing together the several fragments in—(1) All fractures where vicious union produces serious deformity or impairment of function, no matter how long the duration, provided that both these can be corrected. In (2) all fractures where ligamentous union exists in order to secure, either wholly or in part, perfect performance of function. Where such faulty union is caused by intrinsic disease, as syphilis or alcoholism, the chances of success are lessened. In (3) all recent fractures, simple or compound, where muscular action renders difficult the maintenance of the fragments in position. All fractures involving the joints, especially if comminuted, come under this heading. Under all circumstances, the age, physical condition, &c., of the patient must be duly considered. In a valuable paper on the *rôle* of primary and secondary osteoplastic surgery in the treatment of complicated and compound fractures of the extremities, T. H. Manley[13] likewise says that no one should lightly undertake an operation for recent fracture; but when the condition is such as will inevitably be succeeded by marked deformity of the limb, with serious impediment in function, we should never hesitate to open freely and deal with the case as existing conditions require, provided always that the most rigorous asepsis is enjoined. In the treatment of compound fracture, W. G. Le Boutillier[14] truly states that there is less risk of infection in converting a simple into a compound fracture, when we can keep pyogenic bacteria out of the wound, than in suturing muscles and bone in a wound, already, perhaps, so contaminated with bacteria that under the best conditions for resisting inflammation, good circulation, absence of tension, and adequate drainage, infection is more than likely to follow. Exception is of course made for cases where the injury is produced by a clean cutting instrument.

[7] Clin. Jour., May 31, 1899, p. 81. [8] Practitioner, May, 1899, p. 536. [9] Lancet, Oct. 14, 1899, p. 1,026. [10] New York Med. Jour., July 22, 1899, p. 128. [11] Lancet, Aug. 19, 1899, p. 474. [12] Internat. Med. Mag., Aug., 1899. [13] New York Med. Rec., Aug. 19, 1899. [14] Annals of Surg., Sept., 1899, p. 297.

